# Investigating changes in blood-cerebrospinal fluid barrier function in a rat model of chronic hypertension using non-invasive magnetic resonance imaging

**DOI:** 10.3389/fnmol.2022.964632

**Published:** 2022-09-02

**Authors:** Charith Perera, Daniele Tolomeo, Rebecca R. Baker, Yolanda Ohene, Alla Korsak, Mark F. Lythgoe, David L. Thomas, Jack A. Wells

**Affiliations:** ^1^Division of Medicine, UCL Centre for Advanced Biomedical Imaging, University College London, London, United Kingdom; ^2^Division of Neuroscience and Experimental Psychology, University of Manchester, Manchester, United Kingdom; ^3^Geoffrey Jefferson Brain Research Centre, University of Manchester, Manchester, United Kingdom; ^4^Centre for Cardiovascular and Metabolic Neuroscience, Neuroscience, Physiology and Pharmacology, University College London, London, United Kingdom; ^5^Neuroradiological Academic Unit, Department of Brain Repair and Rehabilitation, UCL Queen Square Institute of Neurology, London, United Kingdom; ^6^Dementia Research Centre, UCL Queen Square Institute of Neurology, London, United Kingdom; ^7^Wellcome Centre for Human Neuroimaging, UCL Queen Square Institute of Neurology, University College London, London, United Kingdom

**Keywords:** choroid plexus (CP), arterial spin labelling (ASL), hypertension, blood-cerebrospinal fluid barrier (BCSFB), spontaneous hypertensive rat (SHR), MRI, preclinical studies, imaging

## Abstract

Chronic hypertension is a major risk factor for the development of neurodegenerative disease, yet the etiology of hypertension-driven neurodegeneration remains poorly understood. Forming a unique interface between the systemic circulation and the brain, the blood-cerebrospinal fluid barrier (BCSFB) at the choroid plexus (CP) has been proposed as a key site of vulnerability to hypertension that may initiate downstream neurodegenerative processes. However, our ability to understand BCSFB’s role in pathological processes has, to date, been restricted by a lack of non-invasive functional measurement techniques. In this work, we apply a novel Blood-Cerebrospinal Fluid Barrier Arterial Spin Labeling (BCSFB-ASL) Magnetic resonance imaging (MRI) approach with the aim of detecting possible derangement of BCSFB function in the Spontaneous Hypertensive Rat (SHR) model using a non-invasive, translational technique. SHRs displayed a 36% reduction in BCSFB-mediated labeled arterial water delivery into ventricular cerebrospinal fluid (CSF), relative to normotensive controls, indicative of down-regulated choroid plexus function. This was concomitant with additional changes in brain fluid biomarkers, namely ventriculomegaly and changes in CSF composition, as measured by T1 lengthening. However, cortical cerebral blood flow (CBF) measurements, an imaging biomarker of cerebrovascular health, revealed no measurable change between the groups. Here, we provide the first demonstration of BCSFB-ASL in the rat brain, enabling non-invasive assessment of BCSFB function in healthy and hypertensive rats. Our data highlights the potential for BCSFB-ASL to serve as a sensitive early biomarker for hypertension-driven neurodegeneration, in addition to investigating the mechanisms relating hypertension to neurodegenerative outcomes.

## Introduction

The latest figures provided by the World Health Organization estimate that 1.28 billion adults suffer from hypertension (Zhou et al., 2021). Hypertension is an established risk factor for the onset of neurodegenerative conditions such as Alzheimer’s Disease (AD) (Iadecola et al., 2016). However, there is a limited understanding of the complex changes occurring in the brain under systemic hypertension that lead to downstream neurodegeneration. Moreover, as well as building understanding, it is important to also work toward development of biomarkers for the early detection of brain pathology as a consequence of sustained hypertension.

Disruption of the cerebral vasculature has been well established to originate from the effects of hypertension, e.g., impairment of blood brain barrier (BBB) integrity and reductions in cerebral blood flow (CBF) (Wardlaw et al., 2003). However, the responsibility of mediating the complex interplay between blood and the brain, a key mechanism for central nervous system (CNS) homeostasis, is not limited to only the role of the BBB alone. This responsibility is also attributed to the blood-cerebrospinal fluid barrier (BCSFB) at the choroid plexus (CP)—a relatively understudied barrier within the brain’s cerebrospinal fluid-filled ventricles (Lun et al., 2015) which display a selective vulnerability toward hypertension-driven damage. Prior evidence points to the extent of hypertension-induced damage being more pronounced at the BCSFB than the BBB, which manifests as increased BCSFB leakiness and an altered cerebrospinal fluid (CSF) homeostasis in a manner akin to that observed in AD (Al-Sarraf and Philip, 2003; Castañeyra-Perdomo et al., 2010). The limited study of BCSFB functionality *in-vivo*, due to the reliance on invasive methodologies (Prinzen and Bassingthwaighte, 2000; Hubert et al., 2019), has hindered exploration of pathophysiological changes at the BCSFB that may catalyze neurodegenerative processes, particularly under hypertension.

We have recently developed a translational Magnetic resonance imaging (MRI) technique for the non-invasive assessment of BCSFB function, known as “BCSFB Arterial Spin Labeling (ASL)” (Evans et al., 2020). BCSFB-ASL quantifies the rate of BCSFB-mediated delivery of endogenous arterial blood water to ventricular CSF, thereby providing a surrogate measure of BCSFB function (Evans et al., 2020; Perera et al., 2021). This non-invasive approach may help to better elucidate the BCSFB’s role in the onset of downstream neurodegeneration in the hypertensive brain. Here we present the application of this technique to probe BCSFB functional changes under systemic hypertension, as well as demonstrating the first application in the rat brain.

Spontaneous hypertensive rats (SHR) provide a well-characterized model of human hypertension and present with markers of neurodegeneration such as amyloid-β accumulation, cognitive impairment, cerebral atrophy, BBB dysfunction, and brain fluid dysregulation (Kaiser et al., 2014; Mestre et al., 2018; Wang et al., 2018; Mortensen et al., 2019; Naessens et al., 2020). Using invasive methods, previous studies have detected functional irregularities at the BCSFB locus in the SHR model (Al-Sarraf and Philip, 2003; Castañeyra-Perdomo et al., 2010; Gonzalez-Marrero et al., 2022). Employing a non-invasive standard-ASL and BCSFB-ASL approach, we investigated whether the impairment of BCSFB function is detectable using non-invasive MRI techniques in the SHR model, relative to Wistar Kyoto (WKY) controls. Additionally, we quantified changes in cortical CBF—a conventional biomarker for cerebrovascular health (Hays et al., 2016)—as well as obtaining measures of T1_*CSF*_ and ventricular volumes to assess CSF homeostasis. We hypothesize that non-invasive measures of BCSFB function will reveal its derangement under systemic hypertension.

## Materials and methods

### Animal preparation

All animal procedures were performed under the UK Home Office Act (Scientific Procedures, 1986). SHR and WKY normotensive rats (provided by Envigo) were used in our study (male, *n* = 6 in each group). A pilot study for the optimization of parameters was conducted using the WKY subjects at 10 weeks old. Following parameter optimization, the final data collection was conducted with SHRs and WKYs at 21 weeks old. Body weight measurements at the time of scanning were: WKY 348 ± 5.0 g vs. SHR 369 ± 9.5 g.

Prior to commencing MRI acquisitions, subjects underwent anesthetic induction using 4% isoflurane in 0.8 L/min medical air and 0.2 L/min O_2_. Following induction and weighing, rats were placed into the MRI cradle with bite bar, nose cone and ear bars to ensure a well secured position of the rat head to minimize motion during the data acquisition. Eye ointment was also applied to prevent drying.

A scavenger pump was placed inside the magnet bore to prevent isoflurane build-up. Anesthesia was maintained during the acquisition by reducing isoflurane concentration to 2% in 0.4 L/min room air and 0.1 L/min O_2_.

Temperature and breathing rate were monitored throughout all the experiments using a rectal probe and a respiration pad (SA Instruments). Rat temperature was maintained at 37 ± 0.5^°^C using heated water tubing during data acquisition.

### Magnetic resonance imaging protocols

**FIGURE 1:**
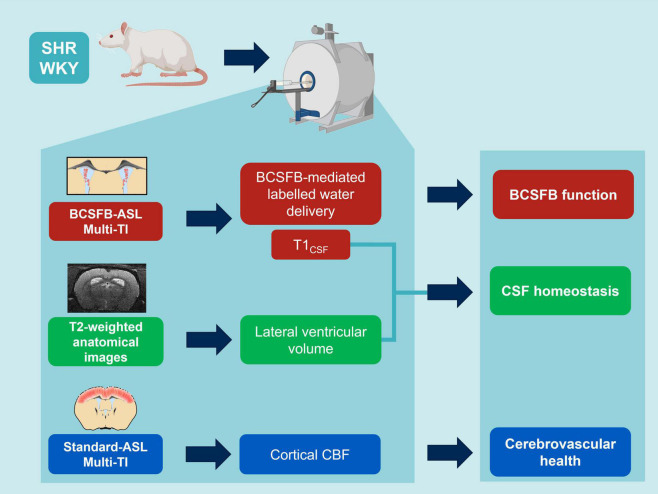
MRI protocols: non-invasive multi-inflow time (multi-TI) arterial spin labeling (ASL) and structural, T2-weighted MRI to inform on hypertension-induced impairment of BCSFB function, CSF homeostasis and cerebrovascular health (created with Biorender.com).

MRI protocols were applied to inform on BCSFB function, CSF homeostasis, and cerebrovascular health (Figure 1). Images were acquired on a 9.4 T Bruker imaging system (BioSpec 94/20 USR) with a horizontal bore and 440 mT/m gradient set with an outer/inner diameter of 205 mm/116 mm, respectively (BioSpec B-GA 12S2), 86 mm volume coil and a four-channel array rat brain surface coil (RAPID Biomedical GmbH) for the transmission and the reception of the RF signal, respectively. The center of the volume coil was positioned caudally 3 cm from the iso-center of the magnet to ensure a good labeling efficiency of the global inversion pulse during the flow-alternating inversion recovery (FAIR)-ASL acquisition.

### Anatomical reference scans

A T2-TurboRARE sequence (fast-spin echo, Paravision v6.0.1) was used to collect sagittal and coronal anatomical reference images to clearly visualize the location of the major CSF compartments in the rat brain. Sequence parameters were: (field of view (FOV) = 30 mm × 30 mm; matrix size = 256 × 256; RARE factor = 8; effective echo time (TE) = 33 ms; repetition time (TR) = 2,500 ms.

Sagittal anatomical reference images (12 × 1 mm slices) were used to position the axial anatomical reference imaging slice and the ASL imaging slices. Coronal anatomical reference images (12 × 0.4 mm slices, 4.8 mm total) were manually positioned to align with the caudal region of the lateral ventricles. These coronal images were subsequently manually segmented to provide an estimate of lateral ventricular volume for each of the subjects.

### Flow-alternating inversion recovery-arterial spin labeling scans using a multi-inflow time approach

Each ASL data set was acquired using a FAIR sequence with a single slice, single shot spin echo—echo planar imaging readout, 20 mm slice-selective width, and a global labeling pulse, across all the experiments.

Parameters for standard-ASL: 2.4 mm slice thickness, matrix size = 32 × 32, FOV = 32 mm × 32 mm, 4 dummy scans, TE = 20 ms. Inflow times (TI) = (200, 500, 1,000, 1,500, 2,000, 3,000, 4,000, 6,000 ms), using TR = 12,000 ms, 5 repetitions per TI.

Parameters for BCSFB-ASL: single slice, 4.8 mm slice thickness, matrix size = 32 × 32, FOV = 32 mm × 32 mm, 6 dummy scans, TE = 220 ms. TI = (200, 750, 1,500, 2,750, 4,000, 5,000, 6,000 ms), using recovery time = 12,000 ms, 10 repetitions per TI.

Importantly, the ASL imaging slice was manually positioned to align with the caudal end of the lateral ventricles, as it has been previously shown to be the predominant region within the lateral ventricles occupied by the CP (Lein et al., 2007). The large slice thickness was chosen to ensure that the slice contained the majority of the lateral ventricles (excluding the more rostral sections which are known to not contain choroid plexus tissue). Therefore, as described in our recent work, our measurements of BCSFB function are concentrated to CP within the lateral ventricles and not the 3rd and 4th ventricles (Evans et al., 2020; Perera et al., 2021).

### Image processing and analysis for relative arterial spin labeling quantification

When analyzing standard-ASL images obtained with TE = 20 ms, a single region of interest (ROI) was drawn for each subject across the cortex of the brain using a non-selective (control) FAIR image, and the mean voxel signal was calculated across the ROI. For each ASL image pair, the non-selective mean ROI value (M_*c*_) was subtracted from the slice-selective (labeled) mean ROI value to provide the perfusion-weighted signal ΔM.

For the BCSFB-ASL technique, our approach was to take the sum of the BCSFB—ASL signal in the lateral ventricles (Supplementary Figure 1). This would enable the measurement of the total amount of labeled arterial-blood-water delivery to ventricular CSF, thereby yielding an overall measure of total BSCFB function in the lateral ventricles. Therefore, for the BCSFB-ASL images, two 3 × 3 voxel ROIs (18 voxels in total, ROI volume = 86.4 mm^3^) were positioned on a slice-selective image, overlaid with the position of the lateral ventricles (Perera et al., 2021). As with the standard-ASL analysis, the combined ROI average signals were subtracted in a pairwise fashion to provide ΔM values, with an added step of subject-wise ventricular volume normalisation to provide volume-normalised, equilibrium magnetization (M0_corr_, equation 1). This correction accounts for the total ventricular volume (quantified from T2-weighted anatomical images) and the ventricular ROI volume (i.e., from the 12 voxels used for the ROI from the low resolution functional data) (Evans et al., 2020). This step is critical for the accurate quantification of the total amount of BCSFB-mediated water delivery; the calculated M0 will be highly dependent on ventricle size due to partial volume effects in the low resolution ASL images (Evans et al., 2020).


$$M\,0_{c\,o\,r\,r}=M\,0\times \frac{T\,o\,t\,a\,l\,v\,e\,n\,t\,r\,i\,c\,u\,l\,a\,r\,v\,o\,l\,u\,m\,e}{V\,e\,n\,t\,r\,i\,c\,u\,l\,a\,r\,R\,O\,I\,v\,o\,l\,u\,m\,e}$$

Equation 1—M0 volume correction for BCSFB-ASL data.

Repeated measures of ΔM and M_*c*_ values were averaged for each TI providing [TI, ΔM] and [TI, M_*c*_] datasets. The [TI, M_*c*_] data were fitted to a simple inversion recovery curve, permitting the extraction of T1 and M0 for each subject. The T1 of the CSF and of cortical brain tissue was calculated from the control images acquired at TE = 220 and 20 ms, respectively. The [TI, ΔM] values were then fit to the relevant Buxton model. The subject-wise T1 (T1_*CSF*_ for BCSFB-ASL or T1_*cortex*_ for standard ASL) and M0 (or M0_*corr*_ for BCSFB-ASL) values extracted from the IR fittings were used in the Buxton model as inputs when calculating CBF and BCSFB-mediated water delivery values for each subject (Evans et al., 2020). For the quantification of cortical CBF, a single-compartment Buxton kinetic model approach was used (Buxton et al., 1998), with BCSFB-ASL requiring a 2-compartment adaptation of this model (Alsop and Detre, 1996; Wang et al., 2002; Evans et al., 2020). The latter adaptation aims to more accurately account for transit effects and intraluminal spins, ultimately allowing for the model to be utilized to describe the delivery of labeled blood water into the CSF (compartment), as opposed to extra-vascular brain tissue in standard-ASL.

The outputs of the model fittings provided subject-wise quantitative values for cortical perfusion (standard-ASL images at TE = 20 ms), and rates of BCSFB-mediated water delivery (BCSFB-ASL images at TE = 220 ms). We report the group average of the individually extracted values of BCSFB-mediated water delivery rate, cortical CBF, and T1_CSF_.

Taking the average rate of BCSFB-mediated labeled water delivery to the lateral ventricles and incorporating the total size of the functional voxels (86.4mm^3^) returns a total BCSFB-labeled water delivery rate to the lateral ventricles.

**FIGURE 2:**
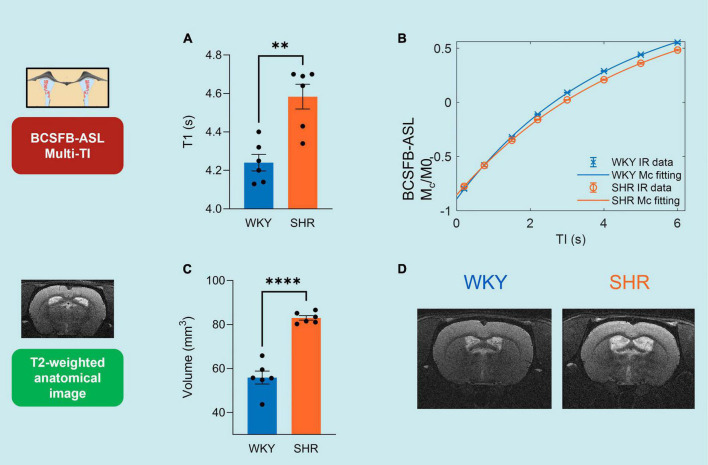
Multi-TI kinetic data for BCSFB-ASL and Standard-ASL. **(A)** Extracted BCSFB-mediated total water delivery in WKY and SHR subjects. **(B)** Averaged BCSFB-ASL multi-TI data alongside kinetic curve fits for WKY and SHR subjects. **(C)** Extracted cortical CBF in WKY and SHR subjects. **(D)** Averaged standard-ASL multi-TI data alongside kinetic curve fits for WKY and SHR subjects. Error bars: ± SEM (*n* = 6 WKY, 6 SHR). Asterisks: 2-tailed *t*-test significance, *p < 0.05, **p < 0.01, ***p < 0.001, and ****p < 0.0001.

For the purpose of visual comparison of the group-averaged kinetic curves (e.g., Figures 2B,D), we averaged the values of ΔM/M0 for standard-ASL, or ΔM/M0_*corr*_ for BCSFB-ASL.

### Pilot study for parameter optimization

The pilot study in 10-week-old WKY controls (*n* = 6) were fit to a 2-compartment Buxton Kinetic model to extract 3 variables: the rate of labeled water delivery, as well as arrival time (Δt) and temporal length (τ) of the tagged bolus of arterial blood water. Based on this pilot dataset, the arrival time (Δt = 1.04 s) and temporal length (τ = 3.66 s) values were fixed when fitting the dataset acquired at 21-weeks to the 2-compartment Buxton kinetic model, in order to extract solely the rate of arterial water delivery with a higher degree of precision (Supplementary Figure 2).

### Measurements of blood pressure

Following completion of the MRI protocols, confirmation of the subjects’ hypertensive state was confirmed through invasive blood pressure measurements in 4 of the subjects within each group (8 in total). Here, an identical anesthesia protocol as used for MRI was employed. Arterial blood pressure was measured through an arterial catheter in the femoral artery and connected to a physiological monitoring system. Data were sampled at 400 Hz for the recording of arterial blood pressure for a baseline reading over 20 min.

### Statistical methods

Statistical comparisons between SHR and WKY data were made using unpaired, 2-tailed, Student’s *t*-tests (GraphPad Prism 9 and Microsoft Excel).

## Results

### Blood-cerebrospinal fluid barrier-mediated water delivery

Recent evidence indicates that the CP-BCSFB system is notably perturbed by systemic hypertension (Al-Sarraf and Philip, 2003; Castañeyra-Perdomo et al., 2010), as such we applied a multi-inflow time (multi-TI) BCSFB-ASL MRI approach in SHR and WKY subjects (*n* = 6 in each group, 21-weeks old) to measure the degree a change in BCSFB derangement under hypertension (Figure 1).

The total rate of BCSFB-mediated labeled arterial water delivery to ventricular CSF was extracted from multi-TI BCSFB-ASL data (Figures 2A,B and Supplementary Figure 4). We measured a 35.8%, decrease in the total rate of water delivery in the SHRs compared to WKYs, which can be observed in the extracted water delivery values WKY 14.4 ± 1.92 μl/min vs. SHR 9.22 ± 1.20 μl/min (*p* = 0.037, 2 -tailed unpaired *t*-test, Figure 2A), as well as from visual inspection of the group-averaged BCSFB-ASL kinetic curves (Figure 2B).

### Cortical cerebral blood flow

Changes in CBF under chronic hypertension can inform on cerebrovascular health. However, CBF changes under hypertension have shown to be variable (Wardlaw et al., 2003; Beason-Held et al., 2007; Li et al., 2015). Here, we applied a multi-TI standard-ASL approach for the extraction of cortical CBF (Figure 2D). Cortical CBF was not significantly different between the WKY normotensive controls and the SHRs, as shown by the group-averaged (*n* = 6) fitted perfusion values: WKY 123 ± 7.4 ml/min/100 g vs. SHR 118 ± 8.7 ml/min/100 g 100 g (*p* = 0.70, Figure 2C). The group-averaged standard-ASL kinetic curves also demonstrate the strong similarity of the two groups (Figure 2D).

### Cerebrospinal fluid homeostasis

Previous reports have shown that SHRs display marked changes in brain fluid management, as determined through measuring perturbations in CSF secretory profiles, glymphatic flow, brain water mobility and ventriculomegaly (Al-Sarraf and Philip, 2003; Castañeyra-Perdomo et al., 2010; Mortensen et al., 2019; Naessens et al., 2020). To further probe how hypertension impacts CSF volume and composition, we acquired measures for surrogate markers of CSF management: T1_*CSF*_ and lateral ventricular volumes.

**FIGURE 3:**
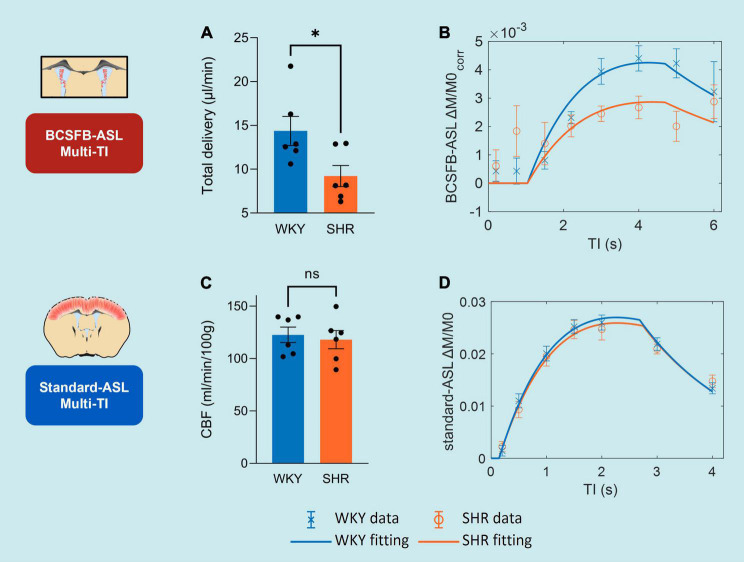
CSF homeostasis: T1_*CSF*_ and lateral ventricular volume. **(A)** T1_*CSF*_ values from WKY and SHR subjects, extracted from BCSFB-ASL multi-TI data. Error bars: ± SEM (*n* = 6 WKY, 6 SHR). **(B)** Example of multi-TI inversion recovery fitting (*n* = 1 WKY, 1 SHR). Error bars: ± stdev (20 repetitions at each TI). **(C)** Lateral ventricular volume values from WKY and SHR subjects, from T2-weighted anatomical images. Error bars: ± SEM (*n* = 6 WKY, 6 SHR). **(D)** Examples of T2-weighted anatomical images (equivalent slices) displaying ventriculomegaly (*n* = 1 WKY, 1 SHR). Asterisks: 2-tailed *t*-test significance, *p < 0.05, **p < 0.01, ***p < 0.001, and ****p < 0.0001.

Single subject examples of data obtained from WKY and SHR groups of the signal (M_*c*_) variation with TI obtained using BCSFB-ASL, alongside the fits to the simple inversion recovery model is depicted in Figure 3B. T1_*CSF*_ was found to be significantly higher in the lateral ventricles of the hypertensive rats: SHR 4.58 ± 0.06 s vs. WKY 4.24 ± 0.04 s (*p* = 0.0012, Figure 3A). Cortical tissue T1 was not significantly different between the two groups: SHR 1.91 ± 0.01 vs. WKY 1.88 ± 0.01 s (*p* = 0.14, data not shown).

Volumes of the lateral ventricles were quantified for all subjects using T2-weighted anatomical images (Figures 3C,D). SHR subjects had a ∼1.5-fold increase in their lateral ventricular volumes relative to normotensive controls: WKY 56 ± 3.0 mm^3^ vs. SHR 83 ± 1.0 mm^3^ (*p* = 6 × 10^–6^, Figure 3C). This is supported by visual inspection of the T2-weighted anatomical images (equivalent slices) from SHR and WKY subjects (Figure 3D).

### Blood pressure measurements

Measurement of blood pressure in our SHR (*n* = 4) and WKY (*n* = 4) rats confirmed their respective blood pressure states (Supplementary Figure 3). SHRs were shown to have a significantly higher blood pressure compared to WKY controls, which was evident from systolic (SHR 185 ± 6.5 vs. WKY 105 ± 12 mmHg, *p* = 0.0010), diastolic (SHR 113 ± 7.5 vs. WKY 55 ± 7.4 mmHg, *p* = 0.0016) and mean blood pressure (SHR 149 ± 6.9 vs. WKY 80 ± 9.5 mmHg, *p* = 0.0011). Additionally, SHRs were found to have an increased blood pressure range (systolic-diastolic) compared to WKYs (SHR 73 ± 2.5 vs. WKY 50 ± 5.4 mmHg, *p* = 0.009).

## Discussion

Patients suffering from systemic hypertension face an increased risk of cognitive decline later in life (Iadecola et al., 2016). Thus, there is a need for not only an improvement in our understanding of disease etiology, but also for early biomarkers of upstream pathological processes. Addressing the need for non-invasive methodologies, we recently developed BCSFB-ASL—a translatable ASL-MRI approach which provides a surrogate measure of BCSFB function by quantifying the rate of labeled endogenous arterial blood-water delivery, mediated by the BCSFB, into ventricular CSF (Evans et al., 2020). In this work we record a 36% reduction in the rate of BCSFB-mediated water delivery in the SHR model relative to normotensive controls, indicating an impaired BCSFB function under chronic hypertension. We also measure perturbations in CSF homeostasis, as measured by changes in T1_*CSF*_ and lateral ventricular volume. However, the cortical CBF measurements revealed no changes under hypertension.

To date, the bulk of work investigating the mechanisms underpinning hypertension-driven neurodegeneration have been centered on studying the cerebral vasculature, revealing impairments to the integrity of the cellular network at the BBB and the associated abnormalities in CBF and cerebrovascular reactivity (Wardlaw et al., 2003; Li et al., 2015). These changes are thought to increase the brain’s vulnerability toward neurodegenerative diseases (Korte et al., 2020; Nielsen et al., 2020). However, despite serving as an established biomarker for neurovascular pathology, there are inconsistencies in the directionality of change for CBF in hypertension studies. Baseline CBF in SHRs has been shown to increase from elevated mean arterial blood pressures (Heinert et al., 1998), but other studies point to CBF decreasing or remaining similar (Grabowski et al., 1993; Lee et al., 2011) due to autoregulatory mechanisms (Li et al., 2015; Naessens et al., 2018; Yazdani et al., 2020). Our multi-TI conventional ASL data revealed no significant difference in the cortical CBF between hypertensive and normotensive groups, likely due to vasoconstrictive autoregulation previously reported in SHRs of a similar age (Li et al., 2015). Our data, combined with the variability in the literature, suggests that CBF measurements may not be the most sensitive biomarker of functional derangement under hypertension.

The BCSFB-ASL methodology was developed and applied first in the mouse brain (Evans et al., 2020). Here, we present the application of BCSFB-ASL under systemic hypertension to investigate functional impairment, as well as the first application of this methodology in the rat brain. In this study, there is a good fit of the multi-TI data to the 2-compartment Buxton Kinetic model, and similar to the initial characterization in the mouse brain, the BCSFB-ASL kinetic curve has features distinct to that of the standard-ASL dataset, i.e., a delayed arrival time for the tagged bolus, delayed time to peak for the ASL signal, and a reduced rate of ASL-signal decay due to the lengthened T1 of lateral ventricular CSF relative to cortical tissue. The healthy rat brain was found to have a total BCSFB-mediated water delivery rate of 14.4 μl/min, which, as expected, is markedly higher than the rate observed in the mouse brain (2.7 μl/min) (Evans et al., 2020), given that the volume of the rat brain is ∼4.5 times larger (Ma et al., 2008; Yang et al., 2021). By providing an initial reference value for BCSFB-mediated water delivery to ventricular CSF in the healthy (WKY) and hypertensive (SHR) groups, we have demonstrated the feasibility of this measurement in larger animal models. In regards to clinical translation, recent work has captured the delivery of labeled blood water into the CSF in the human brain (Petitclerc et al., 2021) using ultra-long TE ASL, and other studies have applied traditional ASL methods to estimate apparent choroid plexus perfusion, with promising results (Johnson et al., 2020, 2021; Zhao et al., 2020).

We detected a marked reduction in rates of BCSFB-mediated water delivery in the SHR providing evidence that the BCSFB-ASL technique can detect derangement of the CP-BCSFB locus in hypertension. The limited study of the CP-BCSFB system *in-vivo* has been due to the lack of non-invasive methodologies, and has historically necessitated the use of radioactive tracers or contrast agents alongside terminal surgical methods (Prinzen and Bassingthwaighte, 2000; Hubert et al., 2019). Given this limitation, the role of the BCSFB in hypertension has only been assessed in *ex-vivo* brain tissues (Al-Sarraf and Philip, 2003; Castañeyra-Perdomo et al., 2010). This literature points to the selective vulnerability of the BCSFB to hypertensive damage, with damage manifesting as a disruption in BCSFB integrity, and an alteration in the profile of proteins secreted into the CSF, which will affect CSF homeostasis and subsequent overall CNS function (Al-Sarraf and Philip, 2003; Castañeyra-Perdomo et al., 2010). Furthermore, hypertensive BCSFB damage was reported to resemble the BCSFB impairment observed with Alzheimer’s Disease (Castañeyra-Perdomo et al., 2010), thus further implicating the BCSFB in the development of dementia. Together, these previous reports support the existence of marked hypertension-induced BCSFB impairment. However, the invasive nature of these methodologies to determine BCSFB impairment has a limited capacity for studies of *in-vivo* disease progression, which our translatable measures of BCSFB function show the potential to provide.

Previous work aiming to investigate how hypertension may modulate BCSFB permeability in the brain has been relatively limited. A total of 42-week-old SHRs were found to display no change in the permeabilities of both the BBB and BCSFB when quantifying fluorescein leakage (Naessens et al., 2018). In contrast, measurements of barrier integrity using ^14^C-sucrose in 12–16 week-old SHRs revealed an increased permeability of the BCSFB (Al-Sarraf and Philip, 2003)., with no difference in BBB permeability detected. The BCSFB-ASL signal, measured here, reflects the average rate of perfusion to the CP convolved with the permeability of the BCSFB to water (the “extraction fraction”; Raichle et al., 1974) and the mass of the CP tissue within the lateral ventricles. Therefore, considering the aforementioned report of increased BCSFB-permeability in the SHR brain, this may suggest that the decreased rates of BCSFB-mediated water delivery detected here primarily reflect decreased CP perfusion. It is important to note, however, that changes in the permeability of the BCSFB to larger tracers may not be indicative of changes in permeability to water (where, for example, Aquaporin-1 is thought to play an important role). Despite the limited specificity of the BCSFB-ASL measurement to changes in BCSFB physiology, it may still represent a valuable biomarker of BCSBF derangement given its non-invasive nature and good reproducibility (Evans et al., 2020) to probe the function of this difficult-to-measure structure.

The SHR model exhibits a range of neurodegenerative changes that occur later in life for patients suffering from chronic hypertension: BBB dysfunction with high levels of amyloid-B accumulation (Wang et al., 2018), non-spatial memory deficits, brain atrophy, neuroinflammation, and dysregulated brain fluid (ISF and CSF) homeostasis (Mortensen et al., 2019). Here, our sensitivity to BCSFB dysfunction suggests that obtaining such *in-vivo* measures may be informative for early detection of the changes that occur in the hypertensive brain that may accelerate the progression of neurodegenerative pathways. Importantly, given the minimal changes in CBF observed in this work, investigation into BCSFB function may provide distinct staging of disease processes relative to more conventional biomarkers of cerebrovascular health. Future studies could perform longitudinal measures of BCSFB function together with imaging biomarkers of neurodegeneration to characterize the timeline and possible predictive relationship of such measurements.

The BCSFB-ASL multi-TI approach also permitted an estimation of changes in T1_*CSF*_ under hypertension, which may arise from alterations in the secretory profile of signaling factors by the CP-BCSFB system that occur concurrently with structural and functional barrier decline (Castañeyra-Perdomo et al., 2010). The changes in CSF management are indicative of brain fluid dysregulation under hypertension which are in line with previous imaging studies, e.g., through abnormalities of glymphatic CSF transport measured with dynamic contrast-enhanced-MRI (DCE-MRI) using the infusion of a gadolinium-based contrast agent into the cisterna magna, in both young (∼8 week-old) and adult (∼20 week-old) SHRs (Mortensen et al., 2019). Imbalance of brain fluid management has also been observed through a reduction in apparent diffusion coefficient (ADC) values in 45-week old SHRs (Naessens et al., 2020), By measuring BCSFB function alongside T1_*CSF*_ changes, this provides the potential for an increased sensitivity to changes in the early phases of hypertension-driven neurodegeneration in a manner that is more translatable than contrast agent infusion into the cisterna magna for DCE-MRI studies of glymphatic function.

Changes in T1_*CSF*_ may stem directly from impairments in BCSFB function in the hypertensive cohort, such as alterations in the secretion of proteins into the CSF. For example, transferrin (Tf), the major iron transport protein in the brain, is secreted by the choroid plexus into the interstitial fluid (ISF) to acquire Fe^3+^, which will then equilibrate with the CSF (Leitner and Connor, 2012; Möller et al., 2019). Relaxivity data obtained from blood serum has shown that Tf and other proteins such as globulins and albumin contribute to the serum T1 relaxation times (Yilmaz et al., 2004). It is likely that the changes in CSF protein composition in hypertensive rats will influence the T1_*CSF*_ values, i.e., potential reductions in CSF Tf-Fe^3+^ levels would lengthen the measured T1_*CSF*_.

Ventriculomegaly in the hypertensive subjects in this work is similar to the increases in volume measured previously in rats of a similar age (Naessens et al., 2020), as well as in hypertensive patients (Salerno et al., 1992; Strassburger et al., 1997; Wiseman et al., 2004). The expansion of this region is reported to arise from a combination of factors including: increase in CSF production rates (Al-Sarraf and Philip, 2003); increased CSF reabsorption resistance; or ventricular expansion due to cerebral atrophy (Naessens et al., 2018). Given that ventriculomegaly is a hallmark of systemic hypertension, this provides an opportunity to investigate the etiology of conditions such as hydrocephalus and dementia, where there is a high degree of clinical overlap and that share hypertension as a common risk factor for their onset.

To conclude, in this work we provide the first application of the BCSFB-ASL technique to the rat brain, providing reference values for rates of BCSFB-mediated water delivery into the lateral ventricles. The decreased rate of BCSFB-mediated arterial water delivery and altered CSF homeostasis in hypertensive subjects demonstrates the potential utility of the method to study the mechanisms that link systemic hypertension to downstream neurodegeneration. The observed functional impairment at the BCSFB suggests that the CP-BCSFB-CSF system may represent a key site of brain vulnerability to systemic hypertension.

## Data availability statement

The raw data supporting the conclusions of this article will be made available by the authors, without undue reservation.

## Ethics statement

The animal study was reviewed and approved by the United Kingdom Home Office Act (Scientific Procedures, 1986).

## Author contributions

CP: conceptualization, data curation, investigation, methodology, formal analysis, and writing—original draft. DT, YO, and RB: methodology and writing—review and editing. AK: data curation and writing—review and editing. ML: conceptualization, resources, and writing—review and editing. DLT: conceptualization, supervision, methodology, and writing—review and editing. JW: conceptualization, supervision, funding acquisition, investigation, methodology, writing—original draft, review and editing, and project administration. All authors contributed to the article and approved the submitted version.

## Supplementary material

Supplementary Figure 1 ROI selection for BCSFB-ASL images. Left: examples of WKY **(A)** and SHR **(B)** ROIs (green) are shown on control (M_*C*_, non-selective FAIR) images (TI = 4 s, *n* = 1 per group). Right: examples of T2-weighted coronal anatomical images for reference (*n* = 1 per group).

Supplementary Figure 2 Pilot data: averaged BCSFB-ASL multi-TI data (WKY, 10-weeks old, *n* = 6). Error bars: ± SEM.

Supplementary Figure 3 Group-averaged arterial pressures from WKY and SHR subjects. Error bars: ± SEM (*n* = 4 WKY, 4 SHR). Asterisks: 2-tailed *t*-test significance, **p* < 0.05, ***p* < 0.01, ****p* < 0.001, and *****p* < 0.0001.

Supplementary Figure 4 BCSFB-ASL multi-TI data: individual subject fits to Buxton kinetic model. **(A)** WKY cohort (*n* = 6). **(B)** SHR cohort (*n* = 6). Error bars: ± standard deviation across 10 repetitions at each TI.
