# Supplementary figures and images for: Investigating changes in blood-cerebrospinal fluid barrier function in a rat model of chronic hypertension using non-invasive magnetic resonance imaging
Source: Front Mol Neurosci. 2022 Sep 2;15:964632. doi: 10.3389/fnmol.2022.964632 (PMC9478509; doi:10.3389/fnmol.2022.964632)

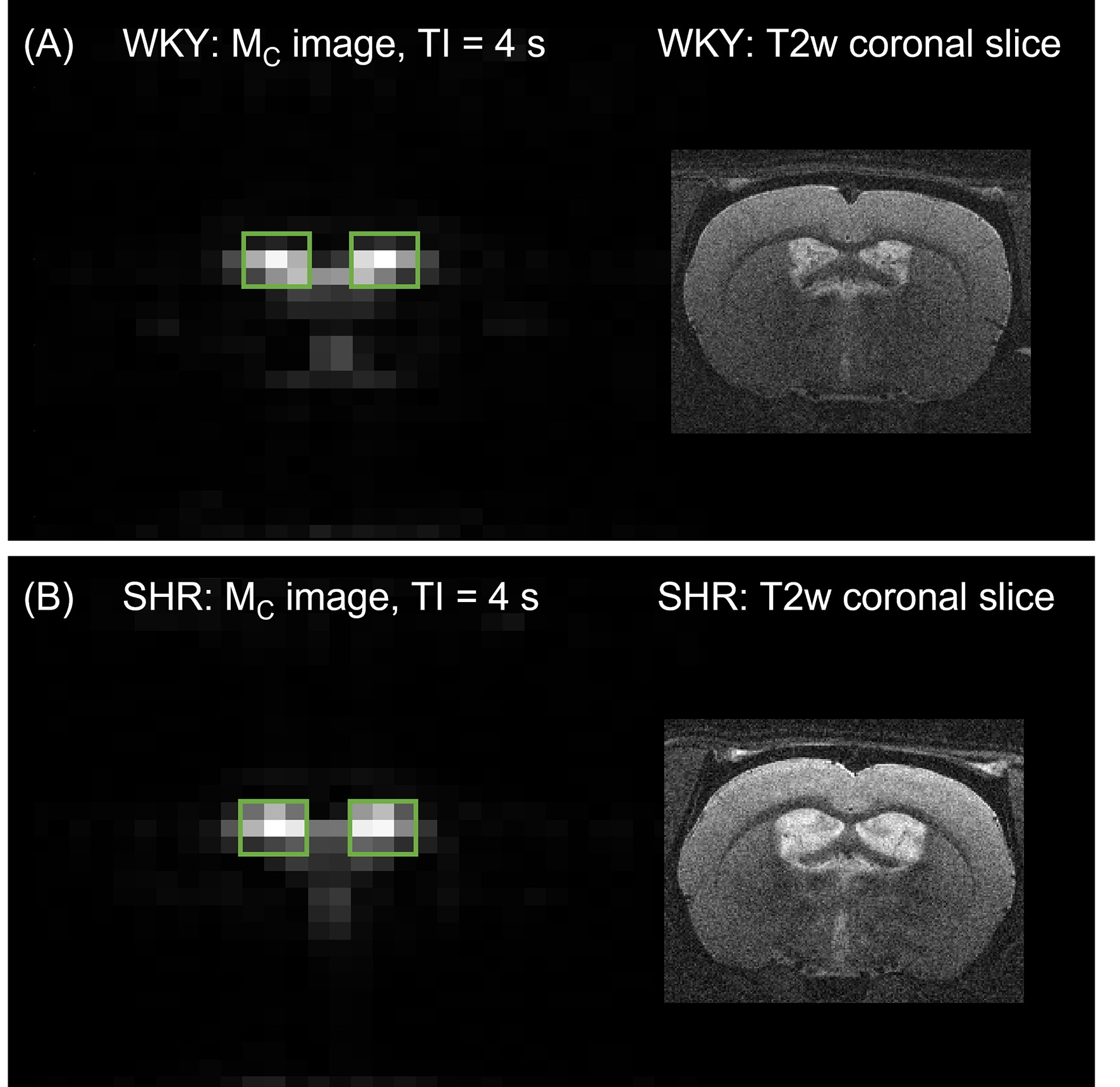

Supplement: Supplementary Figure 1 — ROI selection for BCSFB-ASL images. Left: examples of WKY (A) and SHR (B) ROIs (green) are shown on control (MC, non-selective FAIR) images (TI = 4 s, n = 1 per group). Right: examples of T2-weighted coronal anatomical images for reference (n = 1 per group). [file Image_1.TIF]

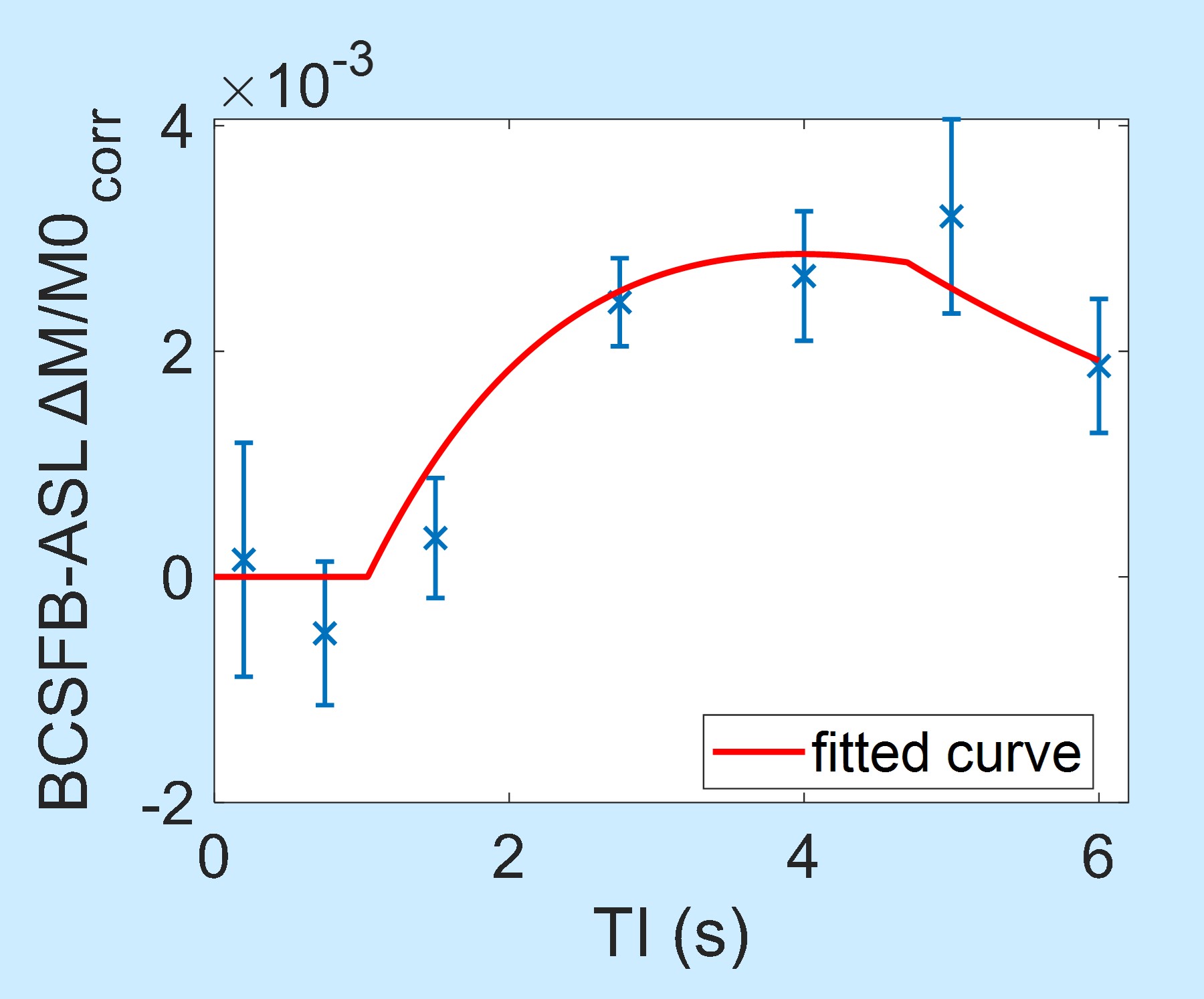

Supplement: Supplementary Figure 2 — Pilot data: averaged BCSFB-ASL multi-TI data (WKY, 10-weeks old, n = 6). Error bars: ± SEM. [file Image_2.jpg]

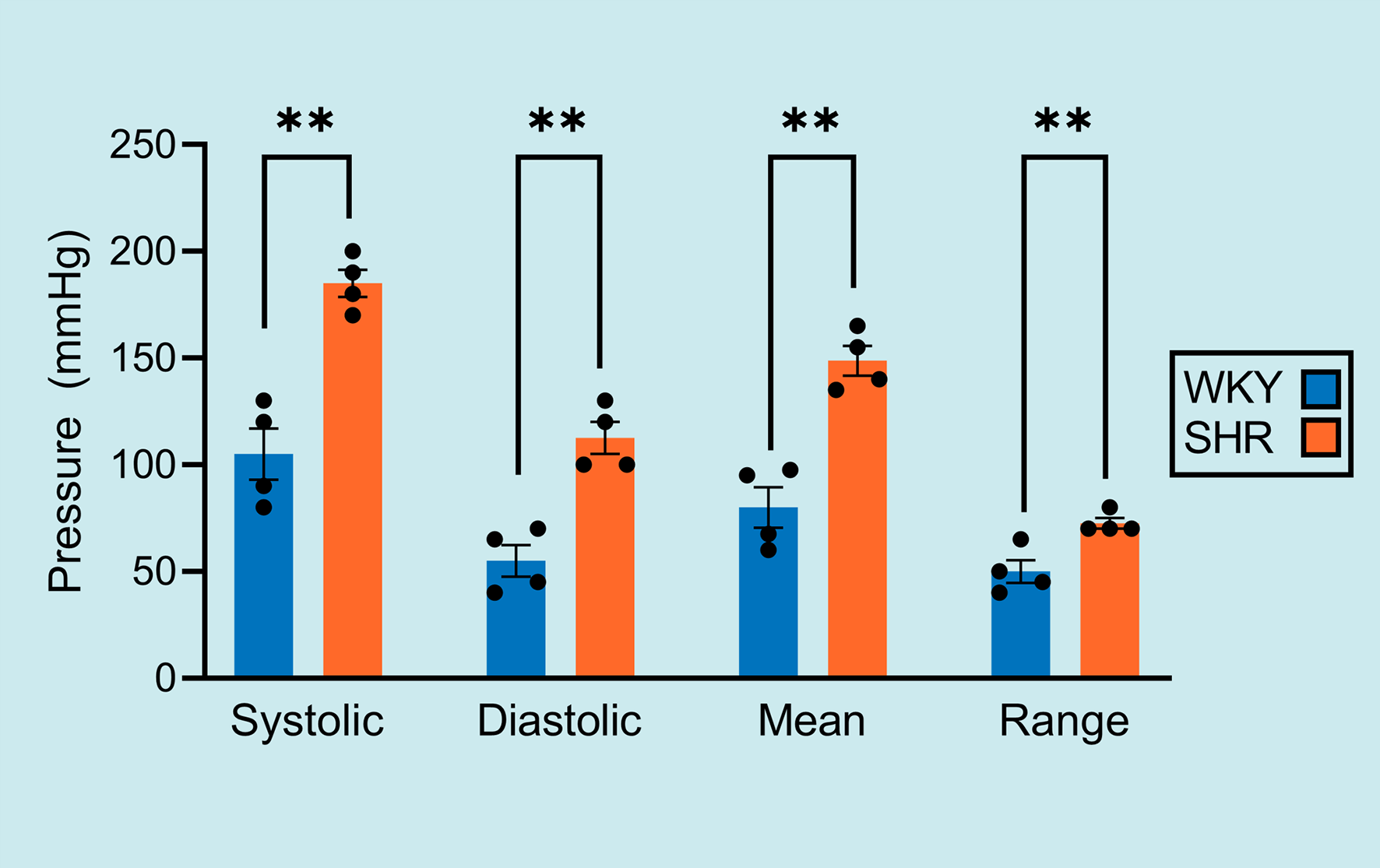

Supplement: Supplementary Figure 3 — Group-averaged arterial pressures from WKY and SHR subjects. Error bars: ± SEM (n = 4 WKY, 4 SHR). Asterisks: 2-tailed t-test significance, *p < 0.05, **p < 0.01, ***p < 0.001, and ****p < 0.0001. [file Image_3.TIF]

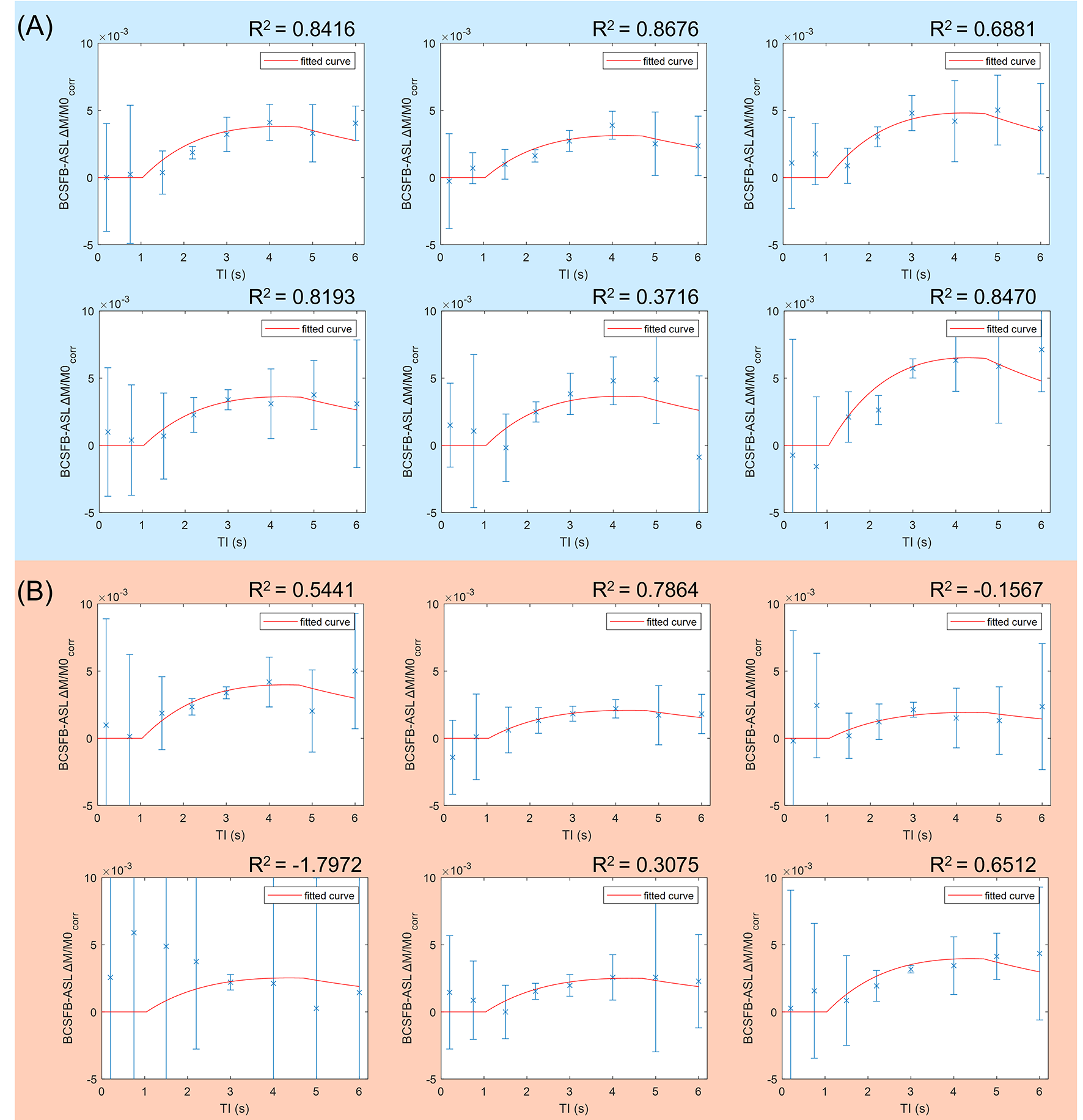

Supplement: Supplementary Figure 4 — BCSFB-ASL multi-TI data: individual subject fits to Buxton kinetic model. (A) WKY cohort (n = 6). (B) SHR cohort (n = 6). Error bars: ± standard deviation across 10 repetitions at each TI. [file Image_4.TIF]
